# Glucose Metabolism Indices and the Development of Chronic Kidney Disease: A Cohort Study of Middle-Aged and Elderly Chinese Persons

**DOI:** 10.1155/2023/1412424

**Published:** 2023-08-02

**Authors:** Lili You, Xiaosi Hong, Qiling Feng, Kan Sun, Diaozhu Lin, Chulin Huang, Chaogang Chen, Chuan Wang, Guojuan Lao, Shengneng Xue, Juying Tang, Na Li, Yiqin Qi, Wanting Feng, Feng Li, Chuan Yang, Mingtong Xu, Yan Li, Li Yan, Meng Ren

**Affiliations:** ^1^Department of Endocrinology, Sun Yat-sen Memorial Hospital, Sun Yat-sen University, 107 Yanjiang West Road, Guangzhou 510120, China; ^2^Guang Dong Clinical Research Center for Metabolic Diseases, 107 Yanjiang West Road, Guangzhou 510120, China; ^3^Department of Clinical Nutrition, Sun Yat-sen Memorial Hospital, Sun Yat-sen University, 107 Yanjiang West Road, Guangzhou 510120, China

## Abstract

**Objective:**

Chronic kidney disease (CKD) has become a major global health issue, and abnormalities of glucose metabolism are a risk factor responsible for development of CKD. We aimed to investigate associations between glucose metabolism indices and CKD in a Chinese population and determine which index is superior for predicting incident CKD.

**Methods:**

We performed a community-based population on 5232 subjects aged ≥40 years without baseline CKD. CKD was defined as an estimated glomerular filtration rate (eGFR) < 60 mL/min/1.73 m^2^ or urinary albumin-to-creatinine ratio (UACR) ≥30 mg/g. We examined the associations of glucose metabolism indices, including fasting plasma glucose (FPG), 2-hour (2 h) oral glucose tolerance test (OGTT), hemoglobin A1c (HbA1c), fasting insulin level, homeostasis model assessment of insulin resistance (HOMA-IR), and HOMA-*β* and the development of CKD.

**Results:**

With an average follow-up of 3.6 years, 6.4% of the subjects developed CKD. Pearson's correlation analysis revealed that FPG, HbA1c, fasting insulin, and HOMA-IR were all significantly correlated with UACR and eGFR. The association persisted in multivariate linear regression analysis adjusted for age and sex. Compared with other glucose indices, HOMA-IR exhibited the strongest associations with CKD in COX multivariate regression analysis (HR = 1.17, 95% CI: 1.04–1.31).

**Conclusion:**

HOMA-IR is superior to other routine indices of glucose metabolism for predicting the development of CKD in middle-aged Chinese persons. Screening with HOMA-IR may help prevent the development of CKD in the general population.

## 1. Background

Chronic kidney disease (CKD) was defined as an abnormality of kidney structure or function that can adversely affect health [1]. CKD has become an important public health problem and is associated with high rates of disability and mortality. In 2016, global years lived with disability among men aged 15 to 49 years with CKD was 0.81% and deaths were 1.94% [2]. The prevalence of CKD (stages 1–5) is estimated to be 3% to 18% globally [3], and around 8.6% for adult males and 9.6% for adult females in high-income countries [4]. A cross-sectional survey based on a nationally representative sample of Chinese adults estimated that around 120 million persons have CKD, with a prevalence of CKD of around 11% [5]. Persons with CKD have a reduced life expectancy due to increased cardiovascular disease and increased all-cause mortality [6]. CKD and associated morbidities are important drivers of increased health care costs [7]. Importantly, in many patients, CKD is not diagnosed until it is in a later stage [8]. Thus, it is important to identify factors that may predict the development of CKD so that early interventions may be given to prevent or delay its development.

The association of glucose metabolism and the development of CKD have been extensively investigated over the past decade. Diabetes is the leading cause of CKD, and up to one-third of adults with newly diagnosed diabetes already have CKD [9]. Diabetes is thought to be responsible for almost 40% of new cases of CKD [10]. Prediabetes also increases the risk of developing CKD [11, 12]. Diabetes and prediabetes diagnosed according to an elevated fasting plasma glucose (FPG) and/or 2-hour oral glucose tolerance test (OGTT) and/or elevated hemoglobins (HbA1c) based on the World Health Organization (WHO) [13] or American Diabetes Association (ADA) [14] criteria are closely related with the development of CKD. Several studies [11, 15, 16] have shown positive associations between CKD and FPG, OGTT, and HbA1c, as well as fasting insulin level, homeostasis model assessment of insulin resistance (HOMA-IR), and HOMA-*β*. A wide body of evidence supports that abnormalities of glucose metabolism are related with the development if CKD. However, associations have mainly been identified through cross-sectional studies; longitudinal data associating indices of glucose metabolisms and CKD are limited. In addition, few studies have examined if any single index of glucose metabolism is superior for predicting the development of CKD.

Thus, the purpose of this study was to use longitudinal data to investigate associations between glucose metabolism indices and CKD in a Chinese population and determine which index is superior for predicting CKD.

## 2. Research Design and Methods

### 2.1. Participants

**Figure 1:**
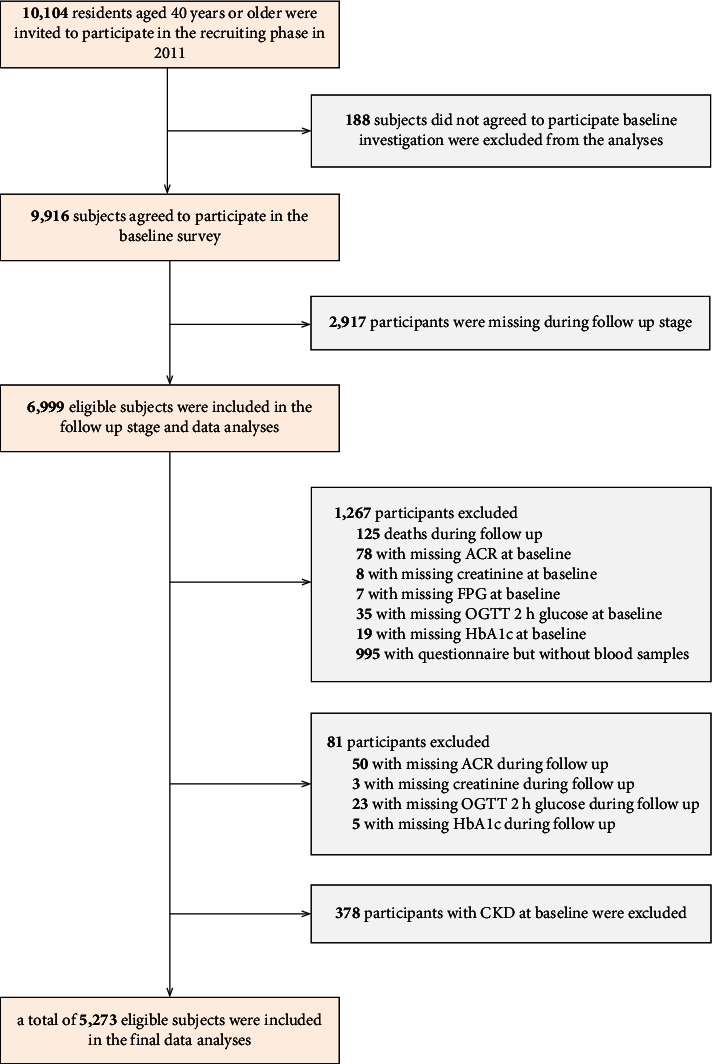
Flowchart of the population selection of the study.

The study population was from the Risk Evaluation of cAncers in Chinese diabeTic Individuals: a IONgitudinal (REACTION) study, which was a multicenter prospective observational study with the aim of evaluating chronic diseases in the Chinese population. Detailed information about the study design and protocols has been published previously [17]. A total of 9916 subjects from a community in Guangzhou, China, signed the informed consent and were included in the baseline survey, from June to November 2011. According to the ADA diagnostic criteria, the baseline diagnosis for the DM group was 1186 cases. All baseline examinations were performed in 2011, and follow-up examinations were carried out from 2014 to 2016. Of the 9,166 individuals included in the baseline survey, 2,917 were lost to follow-up. Thus, a total of 6,999 participants were included in the final dataset (follow-up success rate = 71%). With a mean of 3.6 years follow-up, 125 subjects died and 995 subjects completed the questionnaire by telephone interview. The subjects who failed to provide baseline information of albumin-to-creatinine ratio (ACR) (*n* = 78), creatinine (*n* = 8), FPG (*n* = 7), PPG (*n* = 35), OGTT 2 h glucose (*n* = 35), and HbA1c (*n* = 19) were excluded from the analyses. The individuals who failed to provide follow-up information of ACR (*n* = 50), creatinine (*n* = 3), OGTT 2 h glucose (*n* = 23), and HbA1c (*n* = 5) were excluded from the analyses. For the current study, subjects with baseline CKD were excluded (*n* = 378). Thus, a total of 5,273 individuals were included in the current analyses, which included 1319 people in the DM group and 3954 people in the nondiabetic group. Detailed patient selection for this study is shown in Figure 1.

The study protocol was approved by the Institutional Review Board of Sun Yat-sen Memorial Hospital, Sun Yat-sen University (2014 [2]). The study was performed in accordance with the principles of the Helsinki Declaration, and all participants provided written informed consent.

### 2.2. Clinical and Biochemical Measurement

A standardized questionnaire was used to collect baseline data and was administered by trained interviewers during a face-to-face interview. Information collected included lifestyle factors, sociodemographic characteristics, family history, and the type of diabetes. Current smoking and drinking status were divided into 3 groups: never, ever (the cessation of smoking and drinking for more than half a year), and current (smoking or drinking regularly in the recent half year). The frequency and duration of physical activity were obtained using the International Physical Activity Questionnaire (IRAQ), and the level of physical activity was evaluated by calculating the metabolic equivalent hours per week (MET-h/week). The types of diabetes were divided into 3 groups: type 1 diabetes, type 2 diabetes, other types of diabetes, and the rest. In our study questionnaire, we asked about the types of diabetes and recorded the following responses: 10 cases reported having type 1 diabetes, 241 cases reported having type 2 diabetes, 38 cases reported having other types of diabetes, and the rest were missing data. Notably, although the proportion of type 2 diabetes among diabetes varies by region and year, it generally ranges from 90% to 95% [18–20]. Therefore, in this study, we believe that most of the newly diagnosed diabetic patients in our community-based study were type 2 diabetic patients, especially considering that our study population was aged ≥40 years.

Anthropometrical examinations were conducted by trained staff using standard protocols. Body weight and height were measured with subjects wearing light indoor clothing without shoes and recorded to the nearest 1.0 kg and 0.1 cm, respectively. Body mass index (BMI) was calculated as weight in kilograms divided by height in meters squared (kg/m^2^), and BMI was used to define obesity. Obesity was defined as a BMI ≥ 28.0 kg/m^2^, and overweight when 24.0 kg/m^2^ ≤ BMI < 28.0 kg/m^2^. Waist circumference (WC) was measured at the umbilical level to the nearest 0.1 cm with subjects in the standing position using a nonelastic measuring tape. Central obesity was defined as a WC ≥ 90 cm in men and ≥80 cm in women. Blood pressure was obtained with the subject seated 3 consecutive times at 5 minutes intervals using an automated electronic device (OMRON, Omron Company, Dalian, China). The average of the 3 measurements was used in the analysis. Hypertension was defined as a systolic blood pressure (SBP) ≥ 140 mmHg or a diastolic blood pressure (DBP) ≥ 90 mmHg or the subject reporting that they were receiving regular antihypertensive treatment.

After an overnight fast of at least 10 hours, venous blood samples were collected and stored at 80°C until testing. All patients also received a 2 h OGTT. Measurements of FPG, 2 h OGTT, fasting serum insulin, total cholesterol (TC), triglycerides (TG), low-density lipoprotein cholesterol (LDL-C), high-density lipoprotein cholesterol (HDL-C), aspartate aminotransferase (AST), alanine aminotransferase (ALT), and *γ*-glutamyltransferse (*γ*-GGT) were done using an automated electronic device (Beckman CX-7 Biochemical Autoanalyzer, Brea, California, USA). HbA1c was measured by high-performance liquid chromatography (BioRad, Hercules, CA). HOMA-*β* was calculated using the formula: HOMA-*β* = 20 × (fasting plasma insulin, *μ*U/mL)/(FPG, mmol/L) − 3.5. HOMA-IR was calculated using the formula: HOMA-IR = (FPG, mmol/L) × (fasting plasma insulin, *μ*U/ml)/22.5 [21]. The abbreviated Modification of Diet in Renal Disease (MDRD) formula recalibrated for the Chinese population was used to calculate the estimated glomerular filtration rate (eGFR) expressed as mL/min per 1.73 m^2^. The formula is eGFR = 175 × (serum creatinine × 0.011)^–1.234^ × (age)^−0.179^ × (0.79 if female), with serum creatinine expressed as *μ*mol/L. Diabetes was diagnosed according to the 1999 WHO diagnostic criteria, the level of FPG ≥ 7.0 mmol/L, or the level of 2 h OGTT ≥ 11.1 mmol/L [22].

### 2.3. Definition of Chronic Kidney Disease

CKD was defined according to the latest guidelines of the American Diabetes Association Standards of Medical Care [23]. First morning spot urine samples were collected for determination of the urine albumin-to-creatinine ratio (UACR). Urine albumin was measured by a chemiluminescence immunoassay (Siemens Immulite 2000, United States), and creatinine was measured by Jaffe's kinetic method (Biobase-Crystal, Jinan, China) using an automatic analyzer. UACR was calculated by dividing the urine albumin concentration by the urine creatinine concentrations, and the result was expressed in mg/g. CKD was defined as a UACR ≥ 30 mg/g or an eGFR < 60 mL/min/1.73 m^2^.

### 2.4. Statistical Analyses

Baseline characteristics of study participants were expressed as mean ± standard deviation for continuous variables with a normal distribution, or median and interquartile range (IQR) for continuous variables with a skewed distribution. Categorical variables were summarized as count and percentage. UACR, FPG, 2 h OGTT, HbA1c, fasting insulin, HOMA-IR, HOMA-*β*, TG, ALT, AST, *γ*-GGT, and MET-h/week were logarithmically transformed prior to the analysis due to skewed distributions. Characteristics between groups were compared using one-way ANOVA. Comparisons between categorical variables were performed with the *χ*^2^ test. Correlations between the indices of glucose metabolism (FPG, 2 h OGTT, HbA1c, fasting insulin, HOMA-IR, and HOMA-*β*) with UACR and eGFR were examined with Pearson's correlation analysis and multivariate linear regression. Cox proportional hazard analyses were used to calculate incidence of CKD, and the results were expressed as hazard ratio (HR) and 95% confidence interval (CI). Model 1 was unadjusted; Model 2 was adjusted for age, sex, and BMI; Model 3 was further adjusted for current smoking status, current drinking status, physical activity level, SBP, *γ*-GGT, and LDL-C, which are well-established metabolic factors that may influence glucose metabolism status and CKD progression according to clinical evidence and previous literature. Using continuous variables for logistic regression analysis would answer how much the risk of CKD increases per unit increase in HOMA-IR, but in clinical practice, the significance of HOMA-IR increasing by one unit is negligible. Hence, grouping and comparing by HOMA-IR levels is more clinically relevant. Moreover, quartile categorization evenly divides the data into 4 groups according to sample size, which may minimize the issue of low testing efficiency due to large sample size differences among groups. Finally, we calculated the bin width by dividing the specification tolerance or range (USL-LSL or max-min value) by the number of bins (Bin width = (max-min)/sqrt (*n*)). The first quartile of HOMA-IR is (0.0452, 1.22], *N* = 1318; the second quartile of HOMA-IR is (1.22, 1.73], *N* = 1318; the third quartile of HOMA-IR is (1.73, 2.48], *N* = 1318; and the fourth quartile of HOMA-IR is (2.48, 65.1], *N* = 1318. Based on the abovementioned formula, the bin width of HOMA-IR is 0.03235983, 0.01404793, 0.02065873, and 1.724866 from the first to fourth quartile, respectively. The relations of indices of glucose metabolism with CKD were also examined in subgroups stratified by age (≥58 or <58 years), sex (male or female), degree of obesity categorized by BMI (normal, overweight, or obese), central obesity (yes or no), diabetes (yes or no), and hypertension (yes or no). Interactions were tested by including strata factors, the quartile of glucose metabolism index, and the respective interaction terms (strata factors multiplied by quantiles of glucose metabolism index) simultaneously in the models.

All statistical analyses were performed using SAS software version 9.3 (SAS Institute Inc., Cary, NC, USA). All statistical tests were 2-sided, and values of *P* < 0.05 were considered statistically significant.

## 3. Results

### 3.1. Clinical Characteristics of the Study Population

The 5,273 subjects had a mean age at baseline of 58.7 ± 7.2 years, and after a mean follow-up interval of 3.6 ± 0.7 years, 335 (6.4%) had developed new CKD. The clinical and biochemical characteristics of the subjects at baseline are summarized in Table 1. Compared to those who did not develop CKD, subjects that did had increased FPG, 2 h OGTT, HbA1c, fasting insulin, and HOMA-IR (all *P* < 0.001). However, HOMA-*β* was not different between the 2 groups.

### 3.2. Associations of Baseline Glucose Indices with UACR and eGFR

As shown in Table 2, Pearson's correlation analysis revealed that baseline FPG, 2 h OGTT, HbA1c, fasting insulin, and HOMA-IR were significantly correlated with follow-up UACR and eGFR. No significant relation between HOMA-*β* and UCAR was observed in the correlation analysis. After adjusting for sex and age, a multivariate linear regression analysis showed that the correlations between the baseline glucose indices and UACR persisted (all *P* < 0.001), except for HOMA-*β*. HOMA-IR was most strongly correlated with UACR (*β* = 0.13 and *P* < 0.001) and eGFR (*β* = −0.05 and *P* = 0.0001). Meanwhile, the linear correlation between glucose indices and UACR or eGFR in diabetic and nondiabetic populations separately is shown in Supplementary Tables 1 and 2.

### 3.3. Associations between Baseline Glucose Indices and Risk of CKD

**Figure 2:**
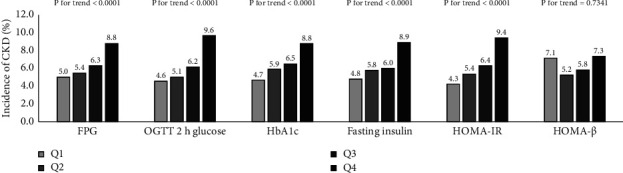
Incidence of chronic kidney disease in different quartiles of baseline glucose indexes.

Figure 2 shows the incidence of CKD in quartiles of the different baseline glucose indices. The incidence of CKD tended to increase with increasing FPG, 2 h OGTT, HbA1c, fasting insulin, and HOMA-IR (all *P* for trend <0.001). No differences of CKD were found in HOMA-*β* quartiles (*P*=0.734). HOMA-IR exhibited the strongest correlations with increased risk of CKD in all of the Cox regression analysis models, indicating that the association between HOMA-IR and CKD was robust and independent (Table 3). The HR for the risk of CKD in the first (lowest) quartile of HOMA-IR was 1.00 (reference); for the second quartile, the HR = 1.15 (95% CI: 0.79–1.69); for the third quartile, the HR = 1.22 (95% CI: 0.84–1.78); and for the fourth quartile (highest), the HR = 1.61 (95% CI: 1.10–2.34).

### 3.4. Subgroup Analyses of HOMA-IR with Risk of CKD

**Figure 3:**
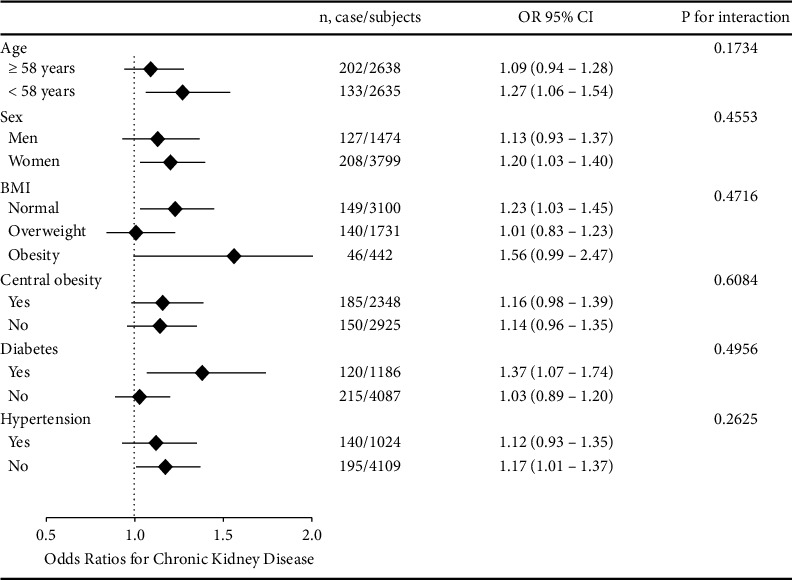
Risk of incident CKD with each quartile increase of HOMA-IR in different subgroups at follow-up. The model is adjusted for age, sex, BMI, current smoking status, current drinking status, physical activity level, SBP, *γ*-GGT, and LDL-C.

As shown in Figure 3, multivariate analyses of subgroups indicated that the association of baseline HOMA-IR with the development of CKD was different in different subgroups. Significant difference of such relationship was detected in subjects with age <58 years, women subjects, those BMI in normal range, those with diabetes, and those without hypertension.

## 4. Discussion

In this study, we evaluated the relations between different indices of glucose metabolism and the development of CKD in a large population of middle-aged Chinese individuals from the REACTION study. The results showed that 3 indices of glucose metabolism, 2 h OGTT, HbA1c, and HOMA-IR, were significantly associated the development of CKD, independent of potential confounding risk factors. Of the 3 indices, HOMA-IR exhibited the best predictive ability. To the best of our knowledge, this is the first and largest population-based cohort study to examine the best index of glucose metabolism for predicting the development of CKD. Because the only effective treatment for ESRD is transplantation, controlling risk factors for the development of CKD and screening methods to determine persons at greater risk of developing CKD are important for decreasing the number of patients developing ESRD. The findings of the present study may assist in identifying persons who are at risk of developing CKD and who may benefit from early interventions.

CKD is becoming a global health problem, and global death rates from kidney diseases have risen by 83% since 1990 [24]. Glucose metabolism has been shown to be an important factor in the development of CKD [12, 25, 26]. Moreover, we performed a subgroup analysis by diabetes status in Figure 3, where we explored the association between HOMA-IR and CKD in diabetic and nondiabetic populations separately. This study indicated that in the diabetic population, after adjusting for confounding factors, the risk of CKD increased significantly with increasing quartiles of HOMA-IR (OR: 1.37 (1.07–1.74)), while in the nondiabetic population, after adjusting for confounding factors, there was also an increasing trend of CKD risk with increasing HOMA-IR levels, OR = 1.03, but it did not reach statistical significance (94% CI: 0.89–1.20). Thus, our finding that an elevated 2 h OGTT and an elevated HbA1c level are independent risk factors for development of CKD is consistent with that of prior studies [26, 27]. Gabir et al. [27] studied 5023 Pima Indian adults, and with a follow-up period of 10 years showed that a 2 h OGTT can predict the development of CKD. Gwang et al. [26] studied 7728 subjects with a median follow-up of 8.7 years; 871 (11.3%) developed CKD and HbA1c was an independent predictor for the development of CKD.

However, there have been conflicting reports of the association of CKD development and glucose metabolism. In a study in Germany, Schöttker et al. [28] followed 3,538 participants during 8 years and found that prediabetes might not contribute to the development of CKD and that preventive efforts such as regular exercise might reduce the risk of developing CKD [29]. An animal study using rats showed that physical training increased insulin sensitivity by enhancing muscle glucose uptake and glucose utilization via glycolysis [30]. A study of hemodialysis patients showed that moderate physical training, using plasma insulin level for patients reduces by 40% [31]. In addition, some cross-sectional studies showed that neither glucose tolerance nor insulin secretion was associated with CKD. Hanssen et al. [32] studied 1796 persons with normal glucose metabolism, 478 with prediabetes and 669 with type 2 DM, and reported no association of CKD with 2 h OGTT or HbA1c. However, the follow-up was relatively short, and the results interpreted as short or intermediate follow-up periods might not capture the associations of 2 h OGTT or HbA1c with CKD. It is possible that there might be geographic or race variability with respect to the association of 2 h OGTT and HbA1c and the development of CKD, as the study has shown that both are predictors of insulin resistance, which is strongly associated with the development of CKD [33]. Clinical significance of each indicator is expected to be studied in the future and further explored with long-term and multistage longitudinal measures to better define the relationship.

The results of this study showed that HOMA-IR was the strongest predictor of the development of CKD in middle-aged and elderly Chinese persons. The HOMA-IR reflects a pathological state in which target tissues fail to respond normally to the biological effects of insulin and is generally considered as an important influential factor for development of CKD. In this study, we investigated the linear correlation between glucose indices and UACR or eGFR in diabetic and nondiabetic populations separately (Supplementary Tables 1 and 2). Besides, we acknowledge that the correlation coefficients between 0.11 and 0.14 represent a relatively low correlation, which may be due to the large sample size of our study, and the wide distribution range of glucose indices and eGFR data, which are not as homogeneous as animal and cell experiments, results in larger correlation coefficients in the linear correlation analysis. The purpose of our correlation analysis was to serve as an exploratory supplementary material, that is, to analyze the same batch of data using multiple statistical methods and to see whether the results of the analysis are consistent, thus increasing the credibility of the article's conclusions. Although the correlation coefficients between 0.11 and 0.14 are relatively low, they all show significant statistical significance, indicating the necessity of further exploring the relationship between HOMA-IR and eGFR. Meanwhile, we selected FPG, OGTT 2 h glucose, HbA1c, fasting insulin, HOMA-IR, and HOMA-*β* as indicators that are commonly used in clinical practice to assess glucose metabolism and insulin resistance. We aimed to explore the association of these glucose metabolism indicators with CKD and compare their predictive value. Therefore, we believe that our analysis of these six indicators is relevant and informative. Meanwhile, this study may provide new ideas and evidence for exploring the mechanism of glucose metabolism and CKD. A study using NHANES data showed that individuals with the highest insulin levels had a 2.65 times greater risk for the development of CKD (95% CI: 1.25–5.62) [34]. Ma et al. [35] studied 3,237 middle-aged and elderly Chinese persons with a 3-year follow-up and showed that an elevated HOMA-IR was associated with accelerated progression of CKD. Huh et al. [36] studied 6,065 Korean persons without CKD at baseline, and over a follow-up period of 10 years showed that insulin resistance was an independent risk factor for development of CKD. Interestingly, the study by Feng et al. also found that the Metabolism Score for Visceral Fat (METS-VF) was a new indicator for predicting CKD risk, which integrated HOMA-IR, waist-to-height ratio, visceral adiposity index, and body mass index [37]. METS-VF can comprehensively evaluate the impact of abdominal adipose tissue on whole-body metabolism, and abdominal adipose tissue is one of the important sources of insulin resistance. Meanwhile, a cross-sectional study conducted by Lin et al. in Southeast China found that HOMA-IR had gender-specific and age-specific associations with albuminuria and renal function impairment, which suggested that HOMA-IR may have different effects on renal function depending on sex hormones and aging [38]. Therefore, different HOMA-IR thresholds may be needed to determine insulin resistance and CKD risk in different gender and age groups. In addition, a prospective study of 73 nondiabetic subjects with CKD showed that of incident CKD in Korean population. However, a prospective study of 73 nondiabetic subjects with CKD showed that HOMA-IR was not significantly different in patients with or without renal endpoints [39]. And the findings of the study might not extend to our study since it was conducted from one region of Turkey with a small sample (*n* = 73).

There are some theories as to why insulin resistance increases the risk of developing CKD. First, normally, insulin binds to the insulin receptor can activate insulin receptor substrate-1 (IRS-1), which can phosphorylated phosphatidylinositol 3-kinase (PI3-K). Under insulin resistance conditions, impaired PI3-K lead to reductions in bioavailable nitric oxide (NO) directly resulting in the development of endothelial dysfunction and CKD [40]. Second, insulin resistance promotes CKD at the molecular level by inflammation through endoplasmic reticulum (ER) stress and is involved in the pathophysiology of chronic kidney injury with tubulointerstitial damage [41]. Moreover, insulin resistance can increase the levels of inflammatory cytokines, which can lead to basement membrane thickening, glomerular mesangial expansion, and the loss of slit pore diaphragm integrity, ultimately leading to glomerulosclerosis and tubule-interstitial injury [42]. Third, insulin resistance may cause overproduction of LDL-C and contribute to hypertriglyceridemia, which can result in renal diseases [43]. Triglyceride-rich apolipoprotein B-containing lipoproteins promote the progression of renal insufficiency [44]. Lastly, insulin resistance promotes CKD by worsening renal hemodynamics through mechanisms such as activation of the sympathetic nervous system, sodium retention, decreased Na^+^, K^+^-ATPase activity, and increased GFR [45, 46].

The causes of insulin resistance are complex and multifactorial and involve genetic factors such as postreceptor signaling defects, an unhealthy lifestyle that includes a lack of physical activity and poor diet which can lead to obesity, obesity, medications, aging, metabolic acidosis, oxidative stress, inflammation, vitamin D deficiency, uremic toxicity, and anemia, as shown by previous human and animal studies. Other comorbid conditions that are strongly associated with insulin resistance are hypertension, diabetes, and hyperlipidemia.

Several limitations of this study need to be addressed. First, by including only middle-aged and elderly Chinese subjects, the results might not apply to different races or a population of younger individuals. Second, our study population was predominantly female; this was partially because we invited person ≥40 years to participate and females are predominant in this age range in China, and women were more likely to participate than men in all surveys [47, 48]. Third, as with any observational study, all confounding factors that may contribute to the development of CKD may not have been included in the models. Fourth, the “gold-standard” for documenting insulin resistance is the euglycemic clamp test. However, the euglycemic clamp test is time-consuming and requires trained personal so it is rarely used in large epidemiological studies. HOMA-IR is a common method used to assess insulin resistance in large epidemiological studies, and it is relatively well-correlated with the euglycemic clamp technique (*r* = 0.88) [49]. Fifth, we defined the CKD based on the first measurements of eGFR and UACR; however, the gold standard is two measurement results. This approach may have reduced the accuracy of our results; however, the results of 1 measurement correlate well with those of 2 measurements and use of 1 measurement is common in large epidemiological studies [50]. Besides, the follow-up rate in this study of 71% was relatively low; however, large epidemiological investigations rare studies can achieve follow-up rate of ≥85% [51, 52]. It is worth noting that the follow-up rate of another REACTION study with a defined 3-year follow-up in Shandong Province, China, from 2012 to 2015 was 77.8%, which was similar to that of our study [35]. Finally, the follow-up time of 3.6 years on average was relatively short to determine the long-term risk of CKD progression based on HOMA-IR or other glucose metabolism indicators. Therefore, our findings need to be validated by studies with longer follow-up periods that can capture more advanced renal outcomes or declining eGFR as markers of progressive CKD. However, our results still suggested that HOMA-IR may be useful to detect early signs of CKD deterioration, such as increased ACR or reduced eGFR, which may have clinical implications for early prevention and management of CKD. Meanwhile, our study provides new ideas and evidence for the subsequent research on the mechanism of the association between glucose metabolism and CKD.

## 5. Conclusion

Our study demonstrated that HOMA-IR is superior to other glucose metabolism indices in predicting the development of CKD in a middle-aged and elderly Chinese population. HOMA-IR may be a useful screening method to determine persons at risk for the development of CKD and who may benefit from early interventions.

## Figures and Tables

**Table 1:** Characteristics of study population at baseline by incident CKD status at follow-up.

	Without CKD	With CKD	*P*
*n* (%)^*∗*^	4938 (93.6)	335 (6.4)	<0.0001
UACR (mg/g)	7.61 (5.57–10.78)	11.10 (7.09–16.78)	<0.0001
eGFR (ml/min per 1.73 m^2^)	103.1 ± 21.2	95.0 ± 24.6	<0.0001
FPG (mmol/L)	5.40 (5.00–5.90)	5.59 (5.10–6.16)	<0.0001
OGTT 2 h glucose (mmol/L)	7.27 (6.09–9.00)	7.99 (6.52–10.43)	<0.0001
HbA1c	5.90 (5.60–6.20)	6.00 (5.70–6.40)	<0.0001
Fasting insulin (*μ*IU/ml)	7.05 (5.20–9.60)	7.90 (5.80–11.10)	<0.0001
HOMA-IR	1.71 (1.21–2.45)	2.08 (1.40–3.01)	<0.0001
HOMA-*β*	74.8 (52.3–106.1)	77.4 (48.2–113.0)	0.9873
Age (years)	55.5 ± 7.0	58.5 ± 8.5	<0.0001
Male (*n* (%))	1347 (27.3)	127 (37.9)	<0.0001
BMI (kg/m^2^)	23.5 ± 3.2	24.6 ± 3.3	<0.0001
WC (cm)	81.1 ± 9.3	84.2 ± 9.3	<0.0001
WC (cm) adjusted sex	81.2 ± 9.1	83.7 ± 9.0	<0.001
SBP (mmHg)	124.8 ± 15.6	132.5 ± 16.7	<0.0001
DBP (mmHg)	74.9 ± 9.7	77.5 ± 9.9	<0.0001
Current smoking (*n* (%))	404 (8.4)	37 (11.3)	0.0667
Current drinking (*n* (%))	156 (3.3)	12 (3.7)	0.6718
TG (mmol/L)	1.25 (0.92–1.81)	1.49 (1.01–2.14)	<0.0001
TC (mmol/L)	5.23 ± 1.24	5.21 ± 1.26	0.7446
HDL-C (mmol/L)	1.34 ± 0.36	1.23 ± 0.33	<0.0001
LDL-C (mmol/L)	3.16 ± 0.95	3.13 ± 0.96	0.6469
ALT (U/L)	13.0 (9.0–17.0)	13.0 (10.0–19.0)	0.3104
AST (U/L)	18.0 (15.0–22.0)	18.0 (15.0–22.0)	0.7884
*γ*-GGT (U/L)	19.0 (14.0–28.0)	21.5 (15.0–29.5)	0.0164
Physical activity (MET-h/week)	22.3 (10.5–46.0)	24.5 (10.5–46.0)	0.7883

Data were means ± SD or medians (interquartile ranges) for skewed variables or numbers (proportions) for categorical variables. ^*∗*^*n* (%) was for the number of incident CKD status at follow-up. *P* values were for the ANOVA or *χ*^2^ analyses across the groups. CKD: chronic kidney disease; UACR: urinary albumin-to-creatinine ratio; eGFR: estimated glomerular filtration rate; FPG: fasting plasma glucose; OGTT: oral glucose tolerance test; HOMA-IR: homeostasis model assessment of insulin resistance; HOMA-*β*: homeostasis model assessment-*β*; BMI: body mass index; WC: waist circumference; SBP: systolic blood pressure; DBP: diastolic blood pressure; TG: triglycerides; TC: total cholesterol; HDL-C: high-density lipoprotein cholesterol; LDL-C: low-density lipoprotein cholesterol; *γ*-GGT: *γ*-glutamyltransferase; MET-h/week: separate metabolic equivalent hours per week.

**Table 2:** Pearson's correlation and multiple regression analyses of baseline glucose indexes associated with UACR and eGFR at follow-up.

	UACR (mg/g)				eGFR			
	*r*	*P*	St. *β*	*P*	*r*	*P*	St. *β*	*P*
FPG	0.12	<0.0001	0.12	<0.0001	−0.09	<0.0001	−0.05	<0.0001
OGTT 2 h glucose	0.12	<0.0001	0.11	<0.0001	−0.07	<0.0001	−0.02	0.0874
HbA1c	0.12	<0.0001	0.11	<0.0001	−0.11	<0.0001	−0.06	<0.0001
Fasting insulin	0.11	<0.0001	0.10	<0.0001	−0.03	0.0218	−0.04	0.0028
HOMA-IR	0.14	<0.0001	0.13	<0.0001	−0.06	<0.0001	−0.05	0.0001
HOMA-*β*	0.02	0.2071	0.01	0.3651	0.05	0.0003	0.01	0.6249

FPG: fasting plasma glucose; OGTT: oral glucose tolerance test; HOMA-IR: homeostasis model assessment of insulin resistance; HOMA-*β*: homeostasis model assessment-*β*; UACR: urinary albumin-to-creatinine ratio; eGFR: estimated glomerular filtration rate. All parameters were logarithmically transformed prior to analysis due to non-normal distributions. *r*: correlation coefficient; St. *β*: standardized regression coefficient; multiple regression analysis is adjusted for age and sex.

**Table 3:** Association between quartiles of baseline glucose indexes and risk of CKD.

		Quartile 1	Quartile 2	Quartile 3	Quartile 4	1-quartile change^#^
FPG	Model 1	1	1.09 (0.78–1.54)	1.27 (0.91–1.78)	1.84 (1.35–2.51)	1.23 (1.12–1.36)
	Model 2	1	1.04 (0.73–1.47)	1.14 (0.81–1.61)	1.47 (1.06–2.04)	1.14 (1.03–1.27)
	Model 3	1	1.10 (0.76–1.58)	1.15 (0.80–1.64)	1.36 (0.96–1.92)	1.11 (0.99–1.23)

OGTT 2 h glucose	Model 1	1	1.13 (0.79–1.61)	1.38 (0.98–1.94)	2.24 (1.63–3.08)	1.32 (1.20–1.47)
	Model 2	1	1.10 (0.77–1.58)	1.20 (0.84–1.70)	1.75 (1.26–2.42)	1.21 (1.09–1.34)
	Model 3	1	1.11 (0.77–1.61)	1.16 (0.81–1.67)	1.52 (1.08–2.14)	1.15 (1.03–1.28)

HbA1c	Model 1	1	1.27 (0.92–1.77)	1.41 (1.01–1.97)	1.97 (1.44–2.70)	1.24 (1.12–1.37)
	Model 2	1	1.14 (0.81–1.59)	1.29 (0.92–1.82)	1.52 (1.09–2.12)	1.15 (1.04–1.28)
	Model 3	1	1.21 (0.86–1.72)	1.29 (0.91–1.84)	1.50 (1.07–2.12)	1.14 (1.02–1.26)

Fasting insulin	Model 1	1	1.23 (0.88–1.73)	1.27 (0.90–1.78)	1.95 (1.42–2.67)	1.24 (1.12–1.37)
	Model 2	1	1.13 (0.80–1.61)	1.12 (0.79–1.60)	1.61 (1.14–2.27)	1.16 (1.04–1.30)
	Model 3	1	1.10 (0.77–1.59)	1.06 (0.73–1.54)	1.39 (0.96–2.00)	1.11 (0.99–1.24)

HOMA-IR	Model 1	1	1.27 (0.89–1.81)	1.52 (1.07–2.15)	2.32 (1.68–3.21)	1.32 (1.20–1.47)
	Model 2	1	1.20 (0.83–1.73)	1.32 (0.92–1.90)	1.89 (1.33–2.70)	1.23 (1.10–1.38)
	Model 3	1	1.15 (0.79–1.69)	1.22 (0.84–1.78)	1.61 (1.10–2.34)	1.17 (1.04–1.31)

HOMA-*β*	Model 1	1	0.72 (0.52–0.99)	0.80 (0.58–1.09)	1.03 (0.76–1.38)	1.02 (0.92–1.12)
	Model 2	1	0.72 (0.52–1.00)	0.79 (0.57–1.09)	0.97 (0.71–1.33)	1.00 (0.90–1.11)
	Model 3	1	0.72 (0.52–1.01)	0.77 (0.55–1.08)	0.96 (0.70–1.33)	1.00 (0.89–1.11)

Data are odds ratios (95% confidence interval). Participants without CKD at follow-up are defined as 0 and with CKD as 1. ^#^All variables were calculated for 1-quartile change of glucose indices. ^*∗*^AUC (95% CI), area under the receiver operating characteristic curve (AUC), and the corresponding 95% confidence intervals (CIs). Model 1 is unadjusted. Model 2 is adjusted for age, sex, and BMI. Model 3 is adjusted for age, sex, BMI, current smoking status, current drinking status, physical activity level, SBP, *γ*-GGT, and LDL-C.

## Data Availability

Main document data and additional unpublished data from the study are available by sending email to youlli@mail.sysu.edu.cn with proper purposes.

## Additional Points

*Patient and Public Involvement*. Patients and/or the public were not involved in the design, or conduct, or reporting, or dissemination plans of this research. *Strengths and Limitations of This Study*. (i) This study was performed in a large population-based cohort of 5232 Chinese subjects. (ii) Finding of this study based on cohort study may be applied to the majority of CKD patients, and HOMA-IR has a better prediction index for development of CKD. (iii) By including only middle-aged and elderly Chinese subjects, the results might not apply to different races or a population of younger individuals. (iv) Our study population was predominantly female; this was partially because we invited person ≥40 years old to participate and females are predominant in this age range in China.

## Supplementary Materials

Supplementary Materials Supplementary table 1: Pearson's correlation and multiple regression analysis of baseline glucose indexes associated with UACR and eGFR at follow-up in diabetic population. Supplementary table 2: Pearson's correlation and multiple regression analysis of baseline glucose indexes associated with UACR and eGFR at follow-up in nondiabetic population.
