# Supplementary material for: Glucose Metabolism Indices and the Development of Chronic Kidney Disease: A Cohort Study of Middle-Aged and Elderly Chinese Persons
Source: Int J Endocrinol. 2023 Aug 2;2023:1412424. doi: 10.1155/2023/1412424 (PMC10412357; doi:10.1155/2023/1412424)
Supplement: Supplementary Materials — Supplementary table 1: Pearson's correlation and multiple regression analysis of baseline glucose indexes associated with UACR and eGFR at follow-up in diabetic population. Supplementary table 2: Pearson's correlation and multiple regression analysis of baseline glucose indexes associated with UACR and eGFR at follow-up in nondiabetic population. [file 1412424.f1.docx]

| **Sup-Table 1 Pearson’s correlation and multiple regression analysis of baseline glucose indexes associated with UACR and eGFR at follow up in diabetes population** | | | | | | | | |
| --- | --- | --- | --- | --- | --- | --- | --- | --- |
|  | UACR (mg/g) | | | | eGFR | | | |
|  | r | P | St. β | P | r | P | St. β | P |
| FPG | 0.11 | 0.0002 | 0.11 | 0.0001 | -0.09 | 0.0033 | -0.09 | 0.0012 |
| OGTT 2h glucose | 0.07 | 0.0181 | 0.06 | 0.0292 | -0.07 | 0.0213 | -0.05 | 0.0456 |
| HbA1c | 0.10 | 0.0006 | 0.10 | 0.0008 | -0.06 | 0.0393 | -0.05 | 0.0649 |
| Fasting insulin | 0.09 | 0.0018 | 0.09 | 0.0020 | 0.05 | 0.1128 | 0.02 | 0.5329 |
| HOMA-IR | 0.12 | < 0.0001 | 0.12 | < 0.0001 | 0.007 | 0.7848 | -0.02 | 0.4762 |
| HOMA-β | 0.003 | 0.9055 | -0.002 | 0.9591 | 0.10 | 0.0003 | 0.08 | 0.0029 |
| FPG, fasting plasma glucose; OGTT, oral glucose tolerance test; HOMA-IR, homeostasis model assessment of insulin resistance; HOMA-β, homeostasis model assessment-β; UACR, Urinary albumin-to-creatinine ratio; eGFR, estimated glomerular filtration rate.  All parameters were logarithmically transformed prior to analysis due to non-normal distributions.  r, correlation coefficient; St. β, Standardized regression coefficient; Multiple regression analysis is adjusted for age and sex. | | | | | | | | |

| **Sup-Table 2 Pearson’s correlation and multiple regression analysis of baseline glucose indexes associated with UACR and eGFR at follow up in non-diabetes population** | | | | | | | | |
| --- | --- | --- | --- | --- | --- | --- | --- | --- |
|  | UACR (mg/g) | | | | eGFR | | | |
|  | r | P | St. β | P | r | P | St. β | P |
| FPG | 0.03 | 0.1089 | 0.02 | 0.1583 | -0.07 | < 0.0001 | -0.03 | 0.0417 |
| OGTT 2h glucose | 0.04 | 0.0169 | 0.02 | 0.1384 | -0.02 | 0.2109 | 0.001 | 0.9362 |
| HbA1c | 0.04 | 0.0084 | 0.02 | 0.2430 | -0.11 | < 0.0001 | -0.07 | < 0.0001 |
| Fasting insulin | 0.09 | < 0.0001 | 0.07 | < 0.0001 | -0.04 | 0.0083 | -0.06 | 0.0002 |
| HOMA-IR | 0.09 | < 0.0001 | 0.07 | < 0.0001 | -0.05 | 0.0006 | -0.06 | < 0.0001 |
| HOMA-β | 0.07 | < 0.0001 | 0.06 | 0.0003 | 0.009 | 0.5575 | -0.03 | 0.0563 |
| FPG, fasting plasma glucose; OGTT, oral glucose tolerance test; HOMA-IR, homeostasis model assessment of insulin resistance; HOMA-β, homeostasis model assessment-β; UACR, Urinary albumin-to-creatinine ratio; eGFR, estimated glomerular filtration rate.  All parameters were logarithmically transformed prior to analysis due to non-normal distributions.  r, correlation coefficient; St. β, Standardized regression coefficient; Multiple regression analysis is adjusted for age and sex. | | | | | | | | |
